# Synergistic Carcinogenesis of HPV18 and MNNG in Het-1A Cells through p62-KEAP1-NRF2 and PI3K/AKT/mTOR Pathway

**DOI:** 10.1155/2020/6352876

**Published:** 2020-10-09

**Authors:** Ying Zhang, Yue Ma, Chao Zhao, Hu Zhang, Yuepu Pu, Lihong Yin

**Affiliations:** Key Laboratory of Environmental Medicine Engineering, Ministry of Education, School of Public Health, Southeast University, Nanjing 210009, China

## Abstract

N-methyl-N´-nitro-N-nitrosoguanidine is a clear carcinogen, increasing evidence that indicates an etiological role of human papillomavirus in esophageal carcinoma. Studies have reported the synergistic effect on environmental carcinogens and viruses in recent years. On the basis of establishing the malignant transformation model of Het-1A cells induced by synergistic of HPV18 and MNNG, this study was to explore the synergistic carcinogenesis of MNNG and HPV. Our research indicated that HPV&MNNG led to a significant increase in the protein-expression levels of c-Myc, cyclinD1, BCL-2, BAX, E-cadherin, N-cadherin, mTOR, LC3II, and p62, with concomitant decreases in p21 and LC3I. HPV18 and MNNG induced accumulation of p62 and its interaction with KEAP1, which promoted NRF2 nuclear translocation. p62 loss prevents growth and increases autophagy of malignant cells by activating KEAP1/NRF2-dependent antioxidative response. In addition, PI3K and p-AKT were stimulated by HPV&MNNG, and PI3K/AKT/mTOR is positively associated with cell proliferation, migration, invasion, and autophagy during malignant transformation. Taken together, MNNG&HPV regulates autophagy and further accelerates cell appreciation by activating the p62/KEAP1/NRF2 and PI3K/AKT/mTOR pathway. MNNG&HPV may improve Het-1A cell autophagy to contribute to excessive cell proliferation, reduced apoptosis, and protection from oxidative damage, thus accelerating the process of cell malignant transformation and leading to cancerous cells.

## 1. Background

Esophageal cancer (EC) is the fourth cause of death of malignant tumors in China and the seventh most common malignant tumor in the world [1], posing a serious threat to human health. EC is an environment-related tumor with multiple factors and multiple stages of development. There are complex interactions between internal and external causes of the development of esophageal cancer. Epidemiological and laboratory data have confirmed that some chemical factors (nitrosamines, mycosin, tobacco, and alcohol), physical factors (coarse and overheated food), nutrient deficiencies, and microbial infections (bacteria, fungi, HPV, herpes simplex virus, epstein-barr virus, and cytomegalovirus) are risk factors for EC [2–5]. Syrjanen et al. [6] firstly reported the characteristic changes of human papillomavirus (HPV) infection in esophageal cancer tissues since 1982, and researchers turn to pay extensive attention to the correlation between esophageal cancer and HPV infection. In the process of exploiting its complex etiology, more evidence shows that HPV infection is related to the occurrence [7] and development of esophageal cancer [8], and HPV may be an important biological factor leading to esophageal cancer.

HPV is a double-stranded DNA virus that eats squamous epithelium and can cause proliferative lesions [9], papilloma, and squamous cell verrucous lesions [10, 11]. Various studies have found that HPV infection significantly increases the risk of esophageal cancer [12, 13]. Molecular and epidemiologic studies have convincingly demonstrated that 50%~80% of esophageal cancer tissues are infected with different HPV subtypes [14], and high-risk HPV infection was found to significantly increase the risk of esophageal cancer [8, 12]. However, the role of HPV infection EC etiology remains controversial that is geographical variation in the rate of HPV infection detected in EC cases [15, 16]. HPV may play an important role only in high-incidence geographic regions [16]. For example, Teng et al. [17] found that HPV is absent from esophageal squamous cell carcinoma in East China, while research pointed that HPV infection may be one of the many factors contributing to the development of ESCC in Northern China [14]. In addition, Cao et al. studied to suggest that HPV infection may play a role in the development of esophageal cancer in Anyang area [18].

Huai'an is a city in Jiangsu Province of China with a high prevalence of esophageal carcinoma [19]. Our preliminary research indicated a high incidence of HPV infection in the esophageal carcinoma specimens from this area [20]. Further, we found that there was a certain correlation between esophageal cancer and HPV18 infection in Huai'an area [21], and there was also a certain risk of exposure to nitrogenous amine in this area [22]. On this basis, we established a cell model that HPV18 and MNNG synergistically promote Het-1A cell malignant transformation. In the process of malignant transformation, HPV&MNNG accelerates the progress of proliferation and cycle, enhances the antiapoptotic ability, improves the invasion and migration ability, and enables cells to have the ability of independent nonanchor growth and tumor formation in nude mice [23]. However, the etiological role of HPV and MNNG in the carcinogenesis leading to ESCC remains unclear. p62, known as p62/sequestosome-1 (p62/SQSTM1), whose accumulation is required for progression of premalignancy to malignancy, and p62/KEAP1/NRF2 pathway commit cells to the malignant fate under conditions where the metabolic and proliferative functions of mTORC1 and c-Myc are activated [24]. Study found that two of the oncoproteins encoded by HPV, E6 and E7, that play a key role in carcinogenesis by interacting with cellular proteins (e.g., p53 and PI3K) involved in the cell cycle, apoptosis, and differentiation [25]. PI3K/AKT/mTOR signaling pathway could regulate apoptosis and autophagy in cancer cells and is molecular targets for cancer therapy [26]. In this study, we plan to identify the changes of the p62/KEAP1/NRF2 pathway and the PI3K/AKT/mTOR pathway to the nitrosamine and HPV synergistically induced esophageal cancer, to explore the possible molecular mechanism of esophageal cancer, and finally to build a scientific and rational control strategies and measures for preventing esophageal cancer in Huai'an area.

## 2. Materials and Methods

### 2.1. Cell Lines and Cell Culture

Human esophageal epithelial cell Het-1A was purchased from the Chinese Academy of Sciences Cell Bank (Shanghai, China). Het-1A cells with stable expression of HPV18 E6E7 genes (Het-1A-E6E7 cells) and control (Het-1A-control cells) were established by our study group. Het-1A-E6E7 and Het-1A-control cells were cultured in complete medium containing 100 U/mL each of penicillin and streptomycin (Invitrogen, USA) and maintained at 37°C in a humidified atmosphere with 5% CO_2_.

### 2.2. Nitrosamine Exposure to Het-1A-E6E7

At 60%-70% confluence, Het-1A-E6E7 and Het-1A-control cells were exposed to 2 mol/L MNNG or untreated for 24 h, once per passage. Then, cells in the 10^th^ passage, (Het-1A-E6E7-10), 20^th^ passage (Het-1A-E6E7-20), and 35^th^ passage (Het-1A-E6E7-35) were examined for the study of molecular mechanisms.

### 2.3. Detection of Cell Function

#### 2.3.1. Cell Viability Assay

Cell proliferation assay was performed using the CCK-8 Cell Proliferation/Viability Assay Kit (7seabiotech, Shanghai, China) as recommended to the manufacturer's protocol.

#### 2.3.2. Flow Cytometry Analysis

Cell cycle was detected by flow cytometry using a cell cycle analysis kit (KeyGEN, Nanjing, China) according to the protocol. The proliferative index was calculated by the formula for (S + G_2_M)/(G_0_G_1_ + S + G_2_M).

Cell apoptosis was detected by flow cytometry using an Annexin V fluorescein isothiocyanate kit (APC/7-AAD) (KeyGEN, Nanjing, China) according to the protocol.

### 2.4. Total RNA Extraction and qRT-PCR

Total RNA was extracted using TRIzol/chloroform (Invitrogen, USA) according to the manufacturer's instruction. After the reverse transcription, amplification was carried out in a total volume of 20 *μ*L containing SYBR Green real-time PCR Master Mix. Primer sequences used are described in supplementary Table S1. Transcription levels were normalized against *β*-actin.

### 2.5. Gene Silencing by Small Interfering RNA (siRNA)

Silencing p62 expression using siRNA (RiboBio, Guangzhou, China) was performed following the manufacturer's instructions. In brief, 35^th^ Het-1A-HPV-MNNG and Het-1A-HPV cells were transfected with p62 siRNA or the negative control at 5 nM using Lipofectamine 2000® (Thermo, Waltham, MA). Fresh Opti-MEM medium (Gibco, USA) was then added for 24-48 h/37°C incubation.

### 2.6. Intracellular Oxidative Damage Measurement

Oxidative damage was detected by nanoscale clusters using ROS detection kit (KeyGEN Bio TECH, China). Cells (1.5 × 10^5^/well) in logarithmic growth phase were inoculated into 24-well plates that were placed sterile circular slides in advance. After 24 hours, the cells were cultured with medium containing 1 mM [Ag (GSH)]^+^ and was cultured for another 15 hours. Then, remove medium, wash three times with PBS, and seal with cleaned glass slides. Finally, cells were observed, photographed, and preserved under confocal fluorescence microscope.

### 2.7. Nuclear Protein Extraction

Nuclear protein extraction protocol was performed with the NE-PER kit (No: P0028, Beyotime Biotechnology) according to the manufacturer's instructions. In short, cell was placed in 1 mL cytoplasmic protein extraction reagent A mixture containing protease and phosphatase inhibitors. The suspension was incubated on ice for 10-15 min. Then, cytoplasmic protein extraction reagent B was added. This mixture was incubated for 1 min, followed by centrifugation at 14,000 × g for 5 min, and the supernatant representing the cytosolic fraction was collected. The pellet was resuspended in 50 microliters of nuclear protein extraction reagent containing protease and phosphatase inhibitors and vortex for 30 sec. Thereafter, the suspension was centrifuged at 14,000 × g for 10 min, and the supernatant was saved as the nuclear protein extract.

### 2.8. Western Blot

Protein was extracted from the cells using RIPA buffer, resolved by SDS–polyacrylamide gels, and then transferred to polyvinylidene difluoride membranes. Primary antibodies against *β*-actin (1 : 1000, Santa Cruz), GAPDH, p62, c-Myc, cyclinD1, p21, BCL-2, BAX, E-cadherin, N-cadherin, LC3, mTOR, Beclin1, KEAP1, and NRF2 (1 : 1000, Abcam, Cambridge, MA) were used. Peroxidase-conjugated secondary antibody (1 : 5000, Santa Cruz) was used. Finally, the antigen–antibody reaction was visualized by enhanced chemiluminescence assay (ECL, Thermo).

### 2.9. Immunoprecipitation

Cells were lysed in IP lysis buffer and protease inhibitors (Sigma, USA), then were normalized using Pierce™ Rapid Gold BCA Protein Assay Kit (No. A53225). Immunoprecipitation was performed using a Thermo Scientific Pierce immunoprecipitation (CO-IP) kit (No. 26149) according to the manufacturer's instructions. Antibody against NRF2 (1: 200, Boston, USA) and KEAP1 (1 : 200, Boston, USA) were added to the lysates and incubated overnight at 4°C, with rabbit or mouse IgG as the control antibody.

### 2.10. Statistical Analysis

Data analysis was performed by the SPSS 20.0 and the GraphPad Prism 7 software; data were described as the mean ± SD from at least three independent experiments. Student's *t*-test and ANOVA analysis were conducted to analysis variables. Differences between groups were considered to be statistically significant at ^∗^*P* < 0.05.

## 3. Results

### 3.1. HPV&MNNG Promotes Het-1A Cell Proliferation, Antiapoptosis, Invasion, Migration, and Autophagy

**Figure 1:**
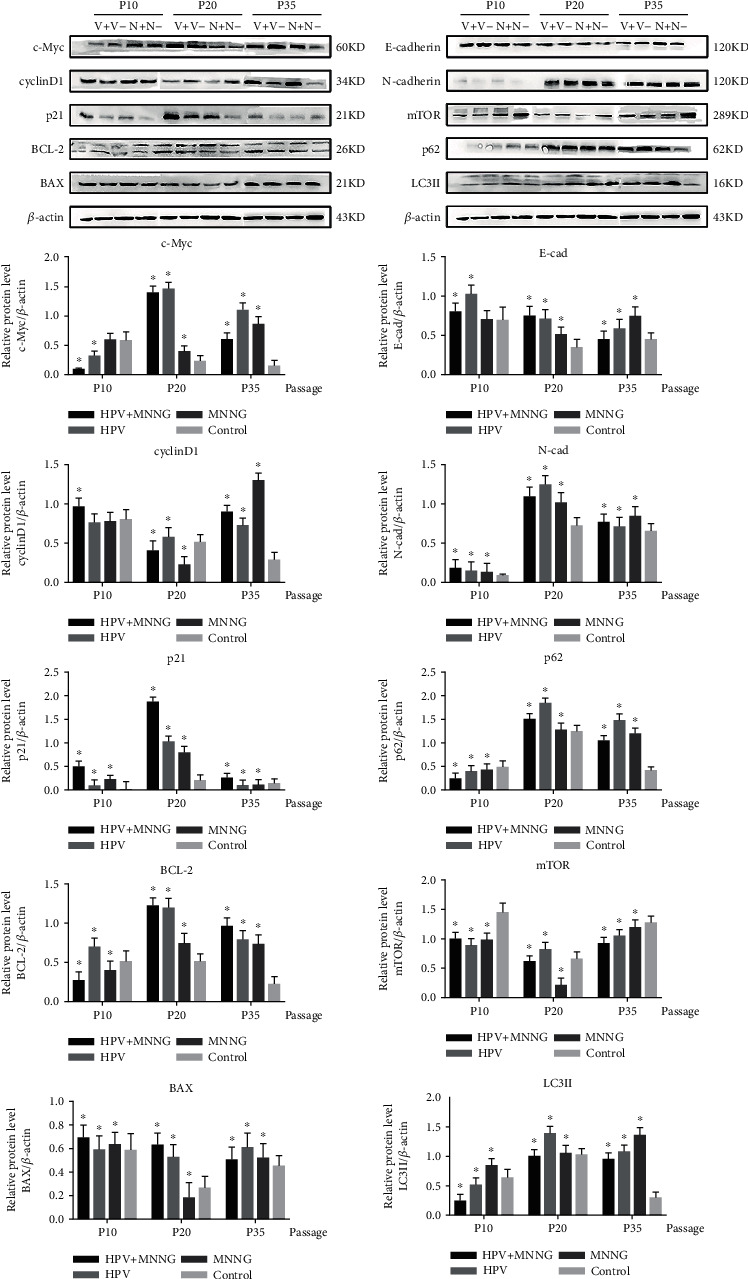
Protein expression of Het-1A during malignant transformation process (V+: HPV&MNNG group; V-: HPV group; N+: MNNG group; N-: control group; P10: 10^th^ passage; P20: 20^th^ passage; P35: 35^th^ passage).

Our published research shows that synergistic of HPV and MNNG caused Het-1A cell malignant transformation via significant enhancement of cell proliferation, antiapoptosis, invasion, migration, and nonanchorage-independent growth [23]. Further, we detected the expression of key protein molecules. In Figure 1, cyclinD1 expression of HPV+MNNG group was first inhibited and then increased, and the expression of cyclinD1 was higher than that in MNNG group of the 35th passage. The c-Myc expression of HPV+MNNG group and MNNG group increased with the infection passage and increased significantly in HPV transfect cells. The p21 expression of four groups was low in 10^th^ and 35^th^ passages, was increased from 20^th^ passages, and was the highest in HPV+MNNG group. The expression of antiapoptotic protein BCL-2 in Het-1A-HPV-MNNG cells and Het-1A-HPV cells were significantly higher than that in the control group. The apoptotic protein BAX of each group increased from the passages, which may be caused by aging. The antiapoptotic ability of Het-1A-HPV-MNNG cell was stronger than that of MNNG group.

In HPV+MNNG group and MNNG group, the E-cadherin expression decreased from the increasing passages, while the expression of N-cadherin protein increased with no significant difference between the two groups. The p62 expression of HPV+MNNG, HPV, and MNNG groups was increased with the increase of algebra during the process of malignant transformation. The mTOR expression of HPV+MNNG group decreased first and then increased, but still lower than that of the control group. The LC3II expression was increased compared with that of the control group, indicating that the combined effect of HPV and MNNG stimulated the occurrence of autophagy at the initial stage.

### 3.2. p62 Loss Prevents Growth of Het-1A-E6E7-MNNG-35 Cells

**Figure 2:**
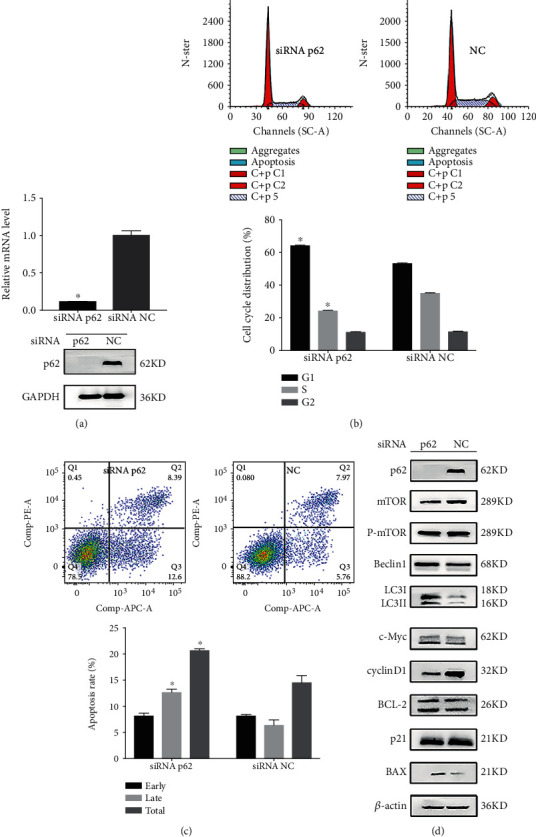
p62 loss prevents growth of Het-1A-E6E7-MNNG-35 cells. (a) p62 mRNA and protein expression in 35^th^ Het-1A-E6E7-MNNG cell after silencing of p62. (b, c) Effect of p62 loss on cell cycle and apoptosis was evaluated by the flow cytometry assay. ^∗^*P* < 0.05. (d) Effect of p62 loss on cell function protein.

Next, we examined whether HPV&MNNG-stimulated p62 upregulation is functionally associated with HPV&MNNG-induced activation of proliferation. Het-1A-E6E7-MNNG-35 cells were transfected with p62 siRNA and NC siRNA (control). As shown in Figure 2(a), p62 expression was significantly inhibited in p62 siRNA-transfected cells compared with control (*P* < 0.05). Then, cell proliferation index was inhibited in p62 siRNA-transfected cells (35.64 ± 0.35%) compared with control siRNA (46.63 ± 0.75%), and cell cycle was blocked at G1 stage (Figure 2(b)). In Figure 2(c), p62 silence promoted apoptosis of Het-1A-E6E7-MNNG-35 cells. In Figure 2(d), p62 silence decreased expression of cyclinD1, but had no effect on p21. The apoptosis-related protein c-Myc was elevated, BCL-2 was downregulated, and BAX was upregulated. Autophagy-related proteins LC3II and Beclin1 were upregulated, and mTOR and p-mTOR were decreased.

### 3.3. p62 Loss Increases Autophagy in Malignant Het-1A Cells

**Figure 3:**
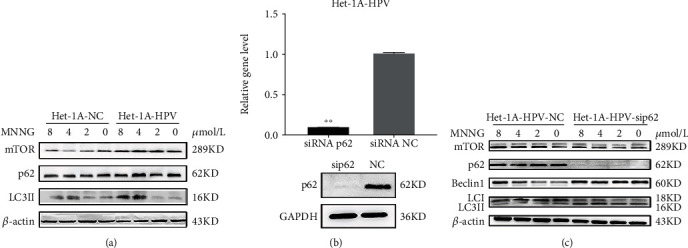
p62 loss increases autophagy in malignant Het-1A cells. (a) Autophagy-related protein expression in Het-1A-HPV and Het-1A-NC cell with MNNG exposure. Het-1A-HPV and Het-1A-NC cells were treated by MNNG at concentrations of 0, 2, 4, and 8 *μ*mol/L for 24 hours. (b) p62 mRNA and protein expression in Het-1A-HPV after silencing of p62. (c) Autophagy-related protein expression in Het-1A-HPV after silencing of p62. Het-1A-HPV-si-p62 and Het-1A-HPV-si-NC were treated with MNNG at concentrations of 0, 2, 4, and 8 *μ*mol/L for 24 hours.

In the process of HPV and MNNG synergistic carcinogenesis, the level of autophagy-related proteins was changed (Figure 1). To further elucidate the effects of HPV, MNNG, and HPV&MNNG on autophagy and the regulatory effect of p62 on autophagy, Het-1A-HPV cells were exposed to MNNG for 24 h at different concentrations (0, 2, 4, and 8 *μ*mol/L). As shown in Figure 3(a), p62 protein and mTOR protein were upregulated mainly by HPV-E6E7, and the expression changes of the two proteins were not obvious with the increase in MNNG concentration. The LC3II was upregulated mainly by MNNG, which means the autophagy function was significantly enhanced (Figure 3(a)). Then, p62 was knocked down in Het-1A-HPV cells (Figure 3(b)), and si-p62-Het-1A-HPV and si-control-Het-1A-HPV were exposed to MNNG for 24 h. We found that mTOR was significantly decreased in the p62 loss group (Figure 3(c)), while Beclin1 and LC3II expression were upregulated by p62 missing (Figure 3(c)). The expression of mTOR protein was positively correlated with p62.

### 3.4. p62 Loss Promotes Malignant Het-1A Cell Autophagy by Activating KEAP1/NRF2-Dependent Antioxidative Response

**Figure 4:**
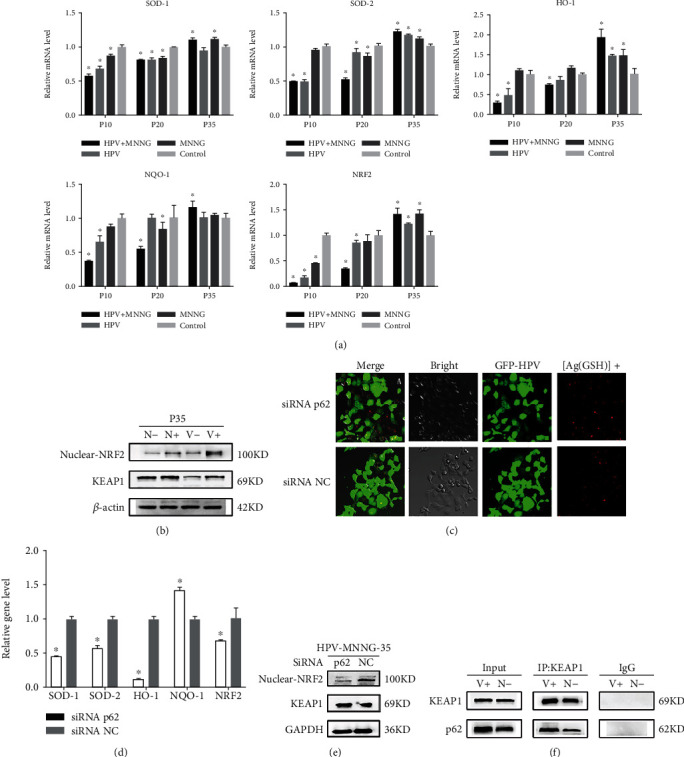
p62 promotes malignant Het-1A cell autophagy by activating KEAP1/NRF2-dependent antioxidative response. (a) mRNA expression of HO-1, NQO-1, and NRF2 in cells at 35^th^ passage (V+: HPV&MNNG group; V-: HPV group; N+: MNNG group; N-: control group). (b) Expression of KEAP1 protein and nuclear protein NRF2 in cells at 35^th^ passage (V+: HPV&MNNG group; V-: HPV group; N+: MNNG group; N-: control group). (c) Oxidative damage of the 35^th^ Het-1A-HPV-MNNG cells after silencing of p62 (green fluorescence was GFP; red fluorescence was the silver nanocluster at the location of the free radical aggregation shown by the excitation at 580 nm). (d) mRNA expression of SOD-1, SOD-2, HO-1, NQO-1, and NRF2 in the 35^th^ Het-1A-HPV-MNNG cells after silencing of p62. (e) Expression of KEAP1 protein and nuclear protein NRF2 in the 35^th^ Het-1A-HPV-MNNG cells after silencing of p62. (f) Interaction between p62 and KEAP1.

**Figure 5:**
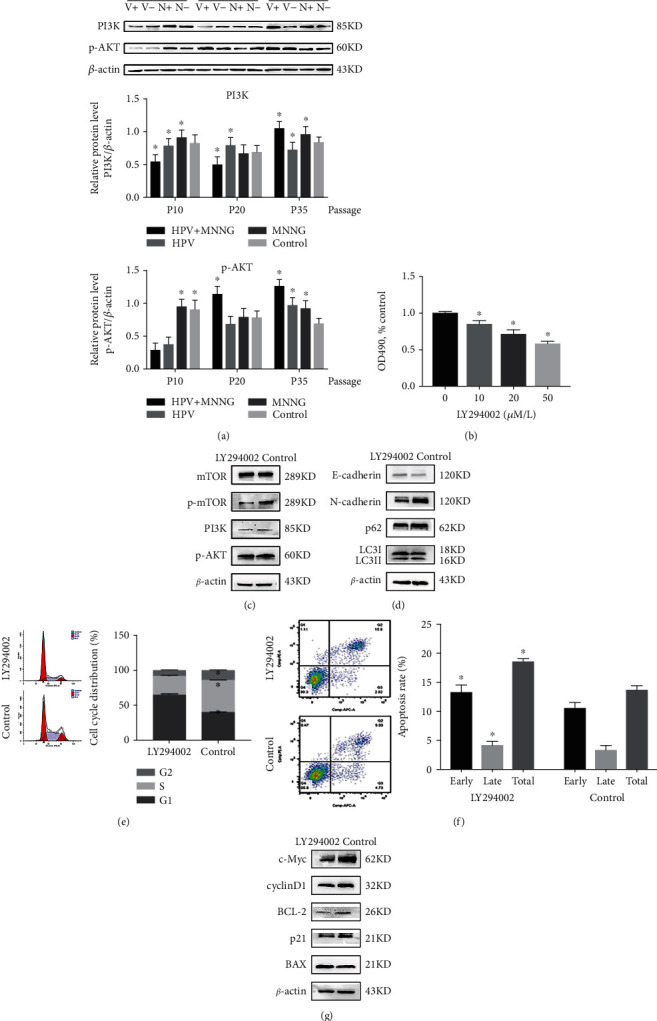
HPV&MNNG activates autophagy of Het-1A cells by the PI3K/AKT/mTOR pathway. (a) Expression of PI3K and p-AKT in cells at 10^th^ passage, 20^th^ passage, and 35^th^ passage (V+: HPV&MNNG group; V-: HPV group; N+: MNNG group; N-: control group). (b) Cell activity of 35^th^ Het-1A-E6E7-MNNG was detected by CCK8 after treatment with LY294002. (c) Protein expression of PI3K/AKT/mTOR pathway after treatment with LY294002. (d) Expression of autophagy-related proteins after treatment with LY294002. (e, f) Het-1A-E6E7-MNNG cell cycle and apoptosis were evaluated by the flow cytometry assay after inhibition of PI3K signal; ^∗^*P* < 0.05. (g) Expression of cell cycle and apoptosis-related proteins after treatment with LY294002.

Moscat et al. [27] pointed out that p62 activates the NRF2-dependent antioxidant response by sequestering KEAP1. In our study, as shown in Figure 4(a), the mRNA expression of SOD-1, SOD-2, HO-1, NQO-1, and NRF2 were decreased in the 10th generation, then recovered gradually in the 20th generation, and finally were increased in in the 35th generation. Among each group, the HPV+MNNG group showed the greatest decrease in the 10th generation and gradually increased, which reached the peak at the 35th generation, indicating that the combined group was more sensitive to oxidative damage stress. After transfection in the 35th generation, the antioxidative damage ability of the malignant cells was enhanced. In Figure 4(b), in 35^th^ passages cells of four groups, the KEAP1 expression was increased in the HPV+MNNG group which was consistent with the level of p62 (Figure 1). NRF2 expression of nuclear was upregulated in the HPV+MNNG group and MNNG group both at protein and mRNA level (Figure 4(b), *P* < 0.05). And NRF2 downstream gene HO-1 was upregulated in HPV+MNNG, HPV, and MNNG groups compared to control (*P* < 0.05, Figure 4(b)). As to NQO-1, there was a peak at HPV group, and level in HPV+MNNG is still higher than control (*P* < 0.05, Figure 4(b)). Moreover, the results of fluorescence confocal microscopy showed that the red fluorescence of the nanoclusters was significantly enhanced after p62 deletion, which indicated that accumulation of ROS increased the oxidative damage in cells. (Figure 4(c); green fluorescence represents GFP; red fluorescence represents the silver nanoclusters where the radicals are excited at the wavelength of 580 NM). With the p62 knocked down, mRNA level of NRF2 (*P* < 0.05, Figure 4(d)) and HO-1 (*P* < 0.01, Figure 4(d)) were significantly downregulated, and NQO1 level was slightly increased (*P* > 0.05, Figure 4(d)). Then, p62 deletion in Het-1A-HPV-MNNG-35 cells resulted in an invariable level of KEAP1 and a decreased level of NRF2 (Figure 4(e)). Finally, we confirmed the interaction between p62 and KEAP1 by coimmunoprecipitation in Het-1A-HPV-MNNG-35 cells. As shown in Figure 5(f), the KEAP1 could be bound to the p62. These data indicate that p62 in Het-1A-HPV-MNNG-35 cells activates NRF2 by regulating KEAP1 abundance; thus, NRF2 inhibited ROS production and promoted tumor development.

### 3.5. HPV&MNNG Activates Autophagy of Het-1A Cells by the PI3K/AKT/mTOR Pathway

As shown in Figure 5(a), expression of PI3K and p-AKT was upregulated during the process of HPV and MNNG synergistic carcinogenesis. The PI3K/AKT signaling pathway is a well-established upstream regulator of mTOR. PI3K/AKT/mTOR is a classical autophagy pathway and also mediates cell survival [28]. As described previously, autophagy level was increased in the process of malignant transformation, and p62 and mTOR are activated to inhibit autophagy (Figure 1). Notably, the trend of the PI3K/AKT pathway to all groups was similar to those of the LC3II and p62 proteins (Figures 1 and 5(a)). To demonstrate whether HPV&MNNG regulates autophagy through the PI3K/AKT/mTOR pathway, we treated PI3K inhibitor LY294002 on 35^th^ Het-1A-HPV-MNNG cells and determined 20 *μ*M as the intervention concentration (Figure 5(b)). Western blot examination indicated that LY294002 treatment downregulated the HPV&MNNG induced increases to p-mTOR, p-AKT, and PI3K, with no change in the total protein levels of mTOR (Figure 5(c)), suggesting the PI3K/AKT/mTOR pathway was inhibited. Autophagy-related proteins LC3II/LC3I ratio (Figure S1B) and p62 were decreased (Figure 5(d)), suggesting the inhibition of autophagy flux. The E-cadherin expression was enhanced, and N-cadherin expression was decreased after inhibition of PI3K, indicating that the EMT of cells was weakened. Then, the flow cytometry analysis showed that cell cycle was arrested in G1 phase, the proliferation index was 35.22 ± 1.02%, which is significantly lower than the control group (60.63 ± 0.30%) (Figure 5(e), *P* < 0.05), and the apoptosis rate (18.62 ± 0.58%) was higher than that of the control group (13.76 ± 0.76%) (Figure 5(f), *P* < 0.05). In addition, cycle-related proteins cyclinD1 and p21 were downregulated, apoptosis-related protein BAX was upregulated, while the antiapoptotic protein BCL-2 was downregulated. The expression of c-Myc, which affects cell proliferation and apoptosis, was significantly decreased (Figure 5(g)). Thus, the promotion of cell proliferation induced by HPV&MNNG was mediated through the activation of PI3K/AKT/mTOR.

## 4. Discussion

### 4.1. Synergistic Carcinogenesis by MNNG and HPV in Het-1A Cell

The paper is aimed at elucidating the synergistic carcinogenic mechanism of environmental carcinogen MNNG and viral HPV on the immortalized human esophageal epithelial Het-1A cell. Carcinogenesis is a multistep process [29]. The fundamental aspects of cancer biology consist of the cell dysregulation of proliferation, cell apoptosis [30], metastasis [31], gene aberration [32], anchorage-independent growth (AIG) [33], and tumor formation. Studies have found that HPV E6 mediates downregulation of p53, leading to DNA damage [34–36]. HPV E7 protein could downregulate the expression of p21 and p27, affect the cell cycle and make cells overproliferate, and result in inducing the occurrence of tumors [34, 36, 37]. In our study, p21 was definitely downregulated. The expression of cyclinD1 showed that cell was cycle arrest first and then promoted from G1 phase to S phase by HPV&MNNG, indicating that cell proliferation capacity went through a stage of inhibition and then enhancement in the process of malignant transformation.

As a vital oncogene, c-Myc promotes tumorigenesis through several mechanisms, including to regulate DNA synthesis, cellular proliferation, differentiation, survival, and immortalization [38–40]. In our study, c-Myc was activated especially by HPV, indicating that HPV played a catalytic role in promoting proliferation and immortalization during the malignant transformation progress. The BCL-2 family proteins (antiapoptotic BCL-2 and proapoptotic BAX) play a central regulatory role in apoptosis [41, 42]. Our result showed that upregulation of BCL-2 and BAX increased BCL-2/BAX ratio (Figure S1A) and promoted cell proliferation in 35^th^ Het-1A-HPV-MNNG.

Epithelial-mesenchymal transformation (EMT) is recognized as the crucial event by which cancer cells acquire an invasive phenotype through the activation of specific transcription factors and signaling pathways [43, 44]. EMT permits cancer cells to acquire migratory, invasive, and distant metastasis [45]. Evidence pointed that reduced N-cadherin and the enhanced E-cadherin expression levels are critical markers for EMT progression [46]. In this study, downregulated E-cadherin and upregulated N-cadherin enhanced invasion and migration ability [23], indicating that the EMT was promoted, which suggested that HPV and MNNG promoted the malignant transformation of Het-1A cell.

To sum up, during the process of malignant transformation, the combined effect of HPV&MNNG showed stronger cell proliferation ability, antiapoptotic ability, invasion, and migration ability.

### 4.2. Synergistic Carcinogenesis of HPV18 and MNNG in Het-1A Cells through p62-KEAP1-NRF2 Signaling Pathway

Autophagy is a fundamental cellular pathway to eukaryotes that function to maintain homeostasis, whose imbalance will lead to the abnormal accumulation of organelles and even the occurrence of tumors [47, 48]. Healthy cells appear to be protected from malignant transformation by proficient autophagy responses [49]. p62 has been described to play diverse biological roles ranging from inflammation to oxidative stress and tumorigenesis [50]. p62, the substrate protein of autophagy, has also been found to play a key role in autophagy [51, 52]. In our study, p62 was upregulated during the cocarcinogenic process of HPV&MNNG on Het-1A cells, as well as expression of autophagy-related proteins mTOR and LC3II (Figure 2). Thus, accumulation of p62 would suggest a blockage in autophagy. Researches show that autophagy mediates oncosuppressive effects accordingly; oncogenic proteins could inhibit autophagy [53].

Therefore, we hypothesized that p62 regulates autophagy as an oncogene in Het-1A-HPV-MNNG cells, which supports the growth, invasion, and metastasis of the tumor. To further validate the role of p62 on malignant transformation and the effect of MNNG and/or HPV on p62, p62 was depleted by shRNA in Het-1A-HPV-MNNG-35 cells and Het-1A-E6E7 cells. Firstly, in Het-1A-HPV-MNNG-35 cells, inhibition of p62 leads to cell cycle arrest, growth inhibition, increased apoptosis rate, and increased autophagy (Figure 3), which means that p62 gene plays an important role in the process of malignant transformation. In Het-1A-E6E7 cells (Figure 4(a)), with high concentrations of MNNG, p62, mTOR, and LC3II were upregulated compare to Het-1A-NC cells. But p62 and mTOR were also upregulated with no MNNG in Het-1A-E6E7 cells, suggesting that HPV led to upregulation of p62 and mTOR. Then, after absence of p62, activity of mTOR was decreased, and the expression of LC3II and Beclin1 was increased. Accordingly, mTOR is positively correlated with p62 expression. Thus, in our study, HPV may activate mTOR signal through p62 protein, leading to autophagy disorder, making high-risk HPV escape the clearance mechanism, promoting the process of malignant transformation. The mammalian or mechanistic target of rapamycin (mTOR) is a serine/threonine kinase that forms two distinct complexes named mTOR complex 1 (mTORC1) and 2 (mTORC2), involving in regulating cell survival, cell growth, cell metabolism, protein synthesis, and autophagy, as well as homeostasis [54–56]. Oncogenic activation of mTOR signaling induces several processes required for cancer cell growth, survival, and proliferation and significantly increases the malignancy of cancer cells [54, 55]. p62 may play its carcinogenic role by enhancing the activation of mTOR. The combined action of HPV and MNNG leads to the disorder of autophagy, the excessive proliferation of cells, and the continuous weakening of antitumor ability.

Oxidative stress is a characteristic of tumor cells, and the KEAP1-NRF2 signaling pathway is the central regulator of the cellular antioxidant process [57]. p62 has KEAP1-binding domain, which could compete with NRF2 for binding KEAP1, which impairs the KEAP1-mediated ubiquitination of NRF2, leading to activation of the NRF2 and promoting the transcription of antioxidant genes [58]. In addition, p62 can also directly interact with NRF2 to ectopic the nucleus, further activating the expression of the downstream antioxidant genes controlled by the antioxidant reaction elements [59]. What more, NRF2 is released from the NRF2-KEAP1 complex by oxidative stress and escapes subsequent degradation and is translocated to the nucleus and activates the expression of target genes [60].

In our study, the mRNA expression of antioxidant factors SOD-1, SOD-2, HO-1, and NQO-1 decreased first and then increased with the transfection algebra, and the expression trends were consistent with that of genes related to p62 and autophagy, and the expression trend of HO-1 and NQO-1 was consistent with that of NRF2. NRF2 nuclear translocation was increased in MNNG+HPV group and MNNG group. After p62 gene silencing, the expression of NRF2 in the nucleus was significantly downregulated, the mRNA levels of SOD-1, SOD-2, and NRF2 and its downstream antioxidant genes HO-1 and NQO-1 were decreased, and the oxidative damage was increased while the ability to resist oxidation was inhibited. Mechanistically, MNNG cooperation with HPV induced compromised autophagy flux, then accumulation of p62 and its interaction with KEAP1, which promoted NRF2 nuclear translocation. Of note, the cell ability against oxidative stress was compromised by p62 gene silencing. Collectively, these findings suggested that HPV synergized with MNNG promotes cellular malignant transformation at least partially via the p62/KEAP1/NRF2 pathway.

### 4.3. Synergistic of HPV18 and MNNG Activates Autophagy of Het-1A Cells by the PI3K/AKT/mTOR Pathway

Aberrations in the regulation of PI3K/AKT signaling and its pathway components have been implicated in tumorigenesis [61]. It has found that PI3K/AKT/mTOR signaling is one of the most important intracellular pathways, which regulates cell growth, motility, survival, metabolism, and angiogenesis [62]. Activation of the PI3K/AKT/mTOR pathway contributes to the development of tumor [63]. In addition, experimental research confirmed the tumorigenic potential for PI3K signal mutations using genetically engineered mouse models (GEMMs) [64, 65]. Pei et al. [66] verified that HO-1 induction could mediate pharmorubicin resistance by promoting autophagy via PI3K/AKT pathway in breast cancer cells. The PI3K/AKT/mTOR pathway has been found to be dysregulated almost in all human cancers, such as breast cancer, colorectal cancer, and hematologic malignancies, which emphasizes the value of targeting this pathway as a potential therapeutic direction in the treatment of cancer [67]. In this study, PI3K/AKT/mTOR pathway was stimulated in the synergistic carcinogenesis process of HPV&MNNG, and PI3K/AKT pathway has similar expression trend to LC3II and p62 proteins. Accordingly, PI3K/AKT signaling might be associated with autophagy during malignant transformation of Het-1A cell. After treated with PI3K inhibitor LY294002, PI3K/AKT/mTOR and autophagy were downregulated in Het-1A-HPV-MNNG cell, as well as inhibited proliferation, promoted apoptosis, and weakened EMT. Thus, in the process of malignant transformation, HPV18 and MNNG activate autophagy of Het-1A cells to accelerate appreciation by the PI3K/AKT/mTOR pathway.

## 5. Conclusion

Taken together, the present study revealed that synergistic effect of MNNG and HPV could promote the malignant transformation of Het-1A cells, including excessive cell proliferation, inhibition of apoptosis, increased migration and invasion, and increased autophagy flux. It was further found that MNNG&HPV activated the P62/KEAP1/NRF2 signaling pathway to increase the transcription of antioxidant genes HO-1 and NQO-1 by upregulating p62 expression, which enhanced the antioxidant ability of cancer cells and further promoted the occurrence and development of tumors. In addition, we elucidated that MNNG&HPV accelerated autophagy flux to promote proliferation and degree of cell malignancy by positively regulating the PI3K/AKT/mTOR pathway.

## Data Availability

The datasets used and/or analyzed during the current study are available from the corresponding author on reasonable request.

## Supplementary Materials

Supplementary Materials Table S1: sequences of the primer for qRT-PCR used in this study. Primer sequences used are described in supplementary Table S1. Transcription levels were normalized against *β*-actin. Figure S1: BCL-2/BAX ratio of Het-1A during malignant process (V+: HPV&MNNG group; V-: HPV group; N+: MNNG group; N-: control group; P10: 10^th^ passage; P20: 20^th^ passage; P35: 35^th^ passage). Figure S1B: LC3II/LC3I ratio in 35^th^ Het-1A-HPV-MNNG cell after inhibition of PI3K.
