# Supplementary material for: Synergistic Carcinogenesis of HPV18 and MNNG in Het-1A Cells through p62-KEAP1-NRF2 and PI3K/AKT/mTOR Pathway
Source: Oxid Med Cell Longev. 2020 Oct 9;2020:6352876. doi: 10.1155/2020/6352876 (PMC7586040; doi:10.1155/2020/6352876)
Supplement: Supplementary Materials — Table S1: sequences of the primer for qRT-PCR used in this study. Primer sequences used are described in supplementary Table S1. Transcription levels were normalized against β-actin. Figure S1: BCL-2/BAX ratio of Het-1A during malignant process (V+: HPV&MNNG group; V-: HPV group; N+: MNNG group; N-: control group; P10: 10th passage; P20: 20th passage; P35: 35th passage). Figure S1B: LC3II/LC3I ratio in 35th Het-1A-HPV-MNNG cell after inhibition of PI3K. [file 6352876.f1.docx]

**Supplementary Materials**

Table S1. Sequences of the primer for qRT-PCR used in this study.

| **Name** | **Chain** | **Sequence (5′ - 3′)** |
| --- | --- | --- |
| GAPDH | F | CATGGGTGGAATCATATTGGA |
|  | R | TCGGAGTCAACGGATTTGG |
| p62 | F | AGATGAGGAAGATCGCCTTG |
|  | R | GGCATCTGTAGGGACTGGAG |
| HO-1 | F | CAGTGCCACCAAGTTCAAGC |
|  | R | GTTGAGCAGGAACGCAGTCTT |
| SOD-1 | F | GGTGTGGCCGATGTGTCTAT |
|  | R | CCTTTGCCCAAGTCATCTGC |
| SOD-2 | F | CTGGACAAACCTCAGCCCTA |
|  | R | CTGATTTGGACAAGCAGCAA |
| NQO1 | F | GGTTTGAGCGAGTGTTCATAGG |
|  | R | GCAGAGAGTACATGGAGCCAC |
| NRF2 | F | CGGTATGCAACAGGACATTG |
|  | R | ACTGGTTGGGGTCTTCTGTG |

Primer sequences used are described in supplementary Table S1. Transcription levels were normalized against β-actin.


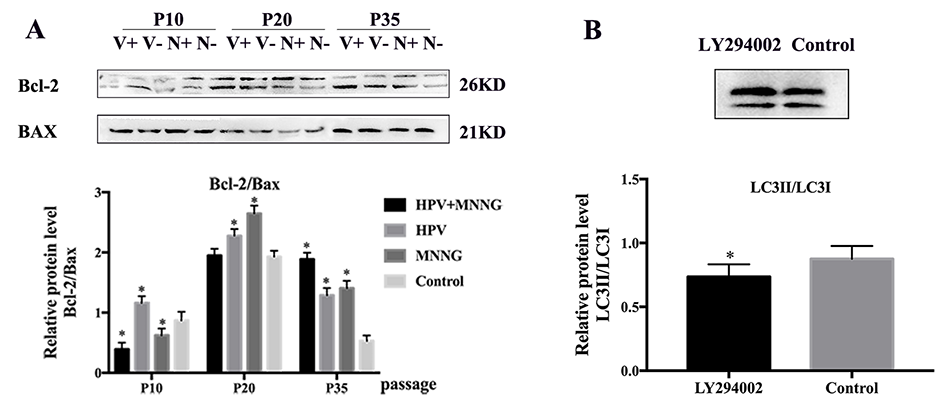


Figure S1. BCL-2/Bax ratio of Het-1A during malignant process. (V+: HPV&MNNG group; V-: HPV group; N+: MNNG group; N-: Control group; P10: 10^th^ passage; P20: 20^th^ passage; P35: 35^th^ passage); Fig. S1B. LC3-II/LC3-I ratio in 35^th^ Het-1A-HPV-MNNG cell after inhibition of PI3K.
